# Properties of Cement-Based Composites Modified with Magnetite Nanoparticles: A Review

**DOI:** 10.3390/ma12020326

**Published:** 2019-01-21

**Authors:** Elżbieta Horszczaruk

**Affiliations:** Faculty of Civil Engineering and Architecture, West Pomeranian University of Technology Szczecin, Al. Piastow 50, 70-311 Szczecin, Poland; elzbieta.horszczaruk@zut.edu.pl; Tel.: +48-91-449-40-59

**Keywords:** cement-based composites, magnetite nanoparticles, mechanical properties, functional properties, hydration products

## Abstract

Despite the many available studies on the evaluation of the influence of nanomaterials on the properties of cement-based composites, the effects of some nanoparticles have not yet been fully recognized. Among the unrecognized nanomaterials are magnetite nanoparticles (MN). The literature devoted to this subject is limited. This paper reviews state-of-the-art research carried out on the effect of MN on the properties of cement-based composites. Detailed descriptions of the processing, microstructures (hydration products), properties (hydration, workability, mechanical and functional properties, and durability), and probability applications of MN-engineered cementitious composites are presented. Particular attention has been paid to MN application methods to the cement composite. Finally, the risks, challenges, and future development of MN-modified cement-based composites is discussed.

## 1. Introduction

Cement composites are commonly used in construction due to their numerous advantages, including high compressive strength, low manufacturing cost, simple production process, and ease of use. However, they have some basic weaknesses: low tensile strength, low resistance to deformation, and susceptibility to cracking [1,2,3]. Ensuring good properties, not only mechanical but also other physico-chemical properties, is a real challenge for present-day material engineers and concrete technologists, particularly in the case of expanded structures, which are often multifactorially prestressed, with complicated shapes and exposed to extreme environmental actions. High-performance and multifunctional cement composites with excellent mechanical properties, durability, and ease of production suitable for the structural material are promising approaches to implementation of cement composite structures in terms of sustainable development [4,5,6].

The greatest and most spectacular achievements in the field of modification of structural materials have been related to the use of nanomaterials. In the construction and building materials industry, nanomaterials have already found commercial application [7,8,9,10,11,12]. The investigation by Colston et al. [13] is considered to be one of the first studies of cement composites modified with nanoparticles (NPs). These investigations showed that incorporation of the nanoparticles into cement leads to significant improvements in cement composites’ microstructures. Later, certain types of the nanomaterials were discovered to not only improve the resistance to brittle cracking and strength of cement composites, but also give them other properties, thus producing multifunctional composites [14,15,16,17]. The nanomaterials used in the building materials industry include silicon dioxide, titanium dioxide, and zinc oxide. These nanomaterials, together with silver nanoparticles, carbon nanotubes, and nanofibers, are most often used for the production of the commercially available building products containing nano-objects. Only a few nanomaterials have been investigated as additions or admixtures to cement concretes and mortars; these include titanium dioxide, aluminum nano oxide, zinc oxide, nano-CaCO_3_, and silicon dioxide [18,19,20,21,22,23]. Most of the research dealing with the modification of cement-based composites concerns titanium dioxide and silicon dioxide, commonly called nanosilica.

Despite the many available studies on the evaluation of the influence of nanomaterials on the mechanical properties of cement composites, the effects of some nanoparticles have not yet been fully recognized. Among the unrecognized nanomaterials are iron oxides (nano-Fe_2_O_3_ and nano-Fe_3_O_4_). The literature devoted to this subject is limited.

Iron oxide (gamma-Fe_2_O_3_), in the form of a nanopowder, has been applied in special anticorrosion coatings, silicones, plastics, rubbers, alloys, lithium batteries, lithium iron phosphate batteries, magnetic seals, wear-resistant materials, and targeted drug delivery. Fe_2_O_3_ is heat-resistant up to a temperature of 450 °C. Above this temperature, the particles lose their magnetic properties. Because the nanoparticle surface area is very large, without surface treatment, the nanoform cannot exist. This means that the nanoparticles cannot produce the nanoproperties. One particle is composed of thousands of single nanoparticles by adsorption of nanoparticles with soft aggregation [24,25].

Fe_3_O_4_ demonstrates high magnetic performance, high saturation magnetization, and low cost. Thus, it has been widely studied as a microwave absorption absorber [26,27]. The main disadvantage of Fe_3_O_4_ is its poor thermal stability, which may lead to the loss of a single domain pole or the special nature of magnetic materials. Therefore, this property restricts their wide application. The protective coatings of the non-metallic materials, e.g., SiO_2_, TiO_2_, and Al_2_O_3_, have been used for improvement of these properties (to increase the thermal stability and diminish oxidation) [28].

In this paper, an overview of the results is presented based on a literature study and our own investigations into the range of the modifications of the cement composites with magnetite nanoparticles (MN). Particular attention has been paid to the nanomaterial application methods to the cement composite. The influence of nanomodification of the structure and the mechanical properties of the cement composites is discussed. The functional properties of the cement-based composites, produced by modification with MN, are also presented.

## 2. Methods of Synthesizing Magnetic Nanostructures

Many studies concerning the synthesis of magnetic nanoparticles were carried out during the recent decades. Significant progress has been achieved in the range of the synthesis, protection, and functionalization of magnetic nanoparticles. The development of such methods as co-precipitation, thermal decomposition and/or reduction, synthesis of micelles, and hydrothermal synthesis has enabled us to control the size and shape of magnetic nanoparticles [29,30]. Efficient methods of magnetic protection against corrosion were developed by surface-active (polymer) coating, silica coating, and carbon coating, or by embedding the magnetic nanoparticles inside the matrix or carrier [31]. The unique shapes of magnetic nanoparticles can be obtained by direct synthesis, in which the anisotropic growth is directed by tuning the reaction conditions or by the use of templates, or by the assembling method, in which a high aspect ratio is achieved by assembly from individual building blocks [32]. Methods including co-precipitation, thermal decomposition and/or reduction, micelle synthesis, hydrothermal synthesis, and laser pyrolysis can all be applied for the synthesis of high-quality magnetic nanoparticles. The advantages and disadvantages of the four main metallic nanostructures’ synthesis methods are summarized in Table 1 [33]. Due to the size and control of the morphology, thermal decomposition seems to be the best synthesis method. Alternatively, microemulsions can be used to synthesize the monodispersion of nanoparticles with various morphologies. The hydrothermal synthesis methods enable the production of high-quality nanoparticles. The co-precipitation and thermal decomposition methods are best known, and enable the production of large amounts of nanomaterial. Detailed information about the synthesis methods has been presented in, among others, Deng et al. [34], Zhang et al. [35], Sun et al. [36], de Mendonça et al. [37], and Fajaroh et al. [38].

**Figure 1:**
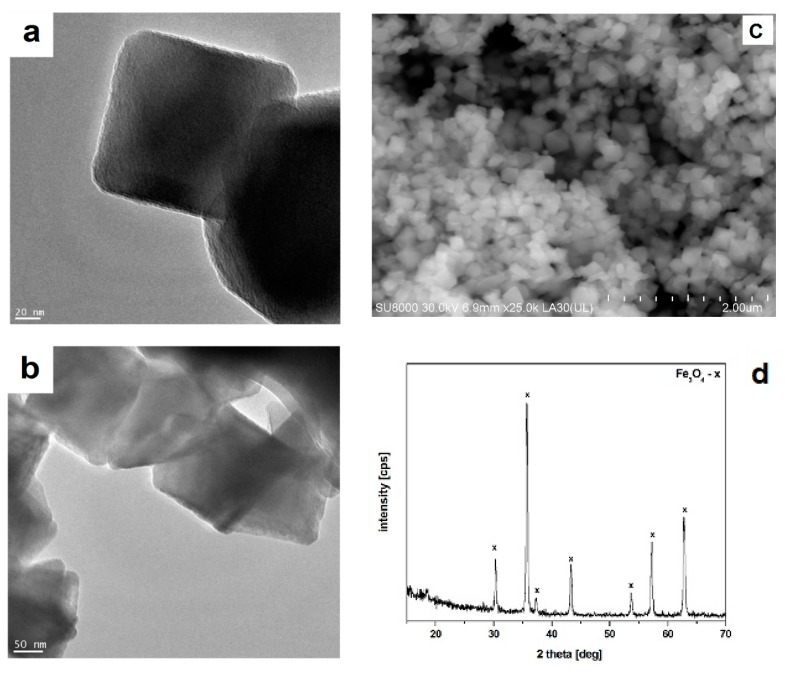
(**a**,**b**) a transmission electron microscopy (TEM) micrograph, (**c**) a scanning electron microscopy (SEM) micrograph, and (**d**) the X-ray diffraction (XRD) pattern of nano-Fe_3_O_4_ [40].

Concrete, as a basic cement-based construction material, should demonstrate—in addition to the physical and mechanical property requirements—resistance to environmental action. Concrete engineering structures should be durable when exposed to high temperature (fire hazard), low temperature (exploitation in winter), and chemical aggression (corrosion of concrete and steel reinforcement). Due to the poor thermal stability of nano-Fe_3_O_4_, when applied to cement composites, the nano-SiO_2_ shell is used for improving the thermal resistance [39]. In Cendrowski et al. [39], the authors attempted to synthesize the magnetite-silica structure of the core-shell type for potential use as a modern admixture designed for modification of building cement composites to improve the properties of the cement composites under high temperature and corrosive environments. The nanostructures with a magnetite (Fe_3_O_4_) core and a solid nano-SiO_2_ shell were used. The commercially available nanomagnetite (Sigma-Aldrich, Darmstadt, Germany) with a diameter of 50–100 nm (Figure 1a–c) was used as the core in the investigation. The X-ray diffraction (XRD) analysis confirmed the occurrence of the characteristic peaks related to the magnetite’s phases (Figure 1d).

**Figure 2:**
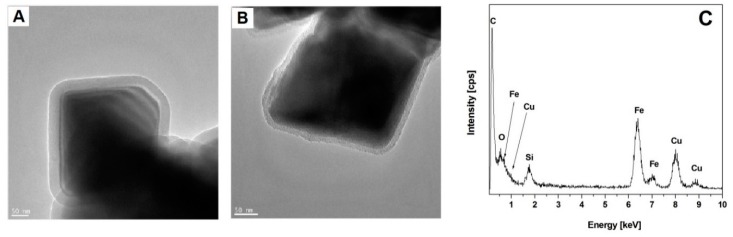
TEM micrographs of (**A**,**B**) iron oxide/silica (Fe_3_O_4_/SiO_2_) core-shell structures (Reprinted with permission from [39]. Cophyright 2017 Elsevier) and (**C**) the energy dispersive X-ray spectrometry (EDS) graph.

Stöber’s method was employed for the synthesis of the silica shell. The thickness of the shell was about 20 nm. After obtaining the nanostructures, the nanomaterial properties were analysed, verifying the efficacy of the synthesis. Then, the chemical stability (in hydrochloric acid) and the thermal stability of the nanocomposites were tested. The transmission electron microscopy (TEM) micrograms of the produced nanostructures are presented in Figure 2 [39].

**Figure 3:**
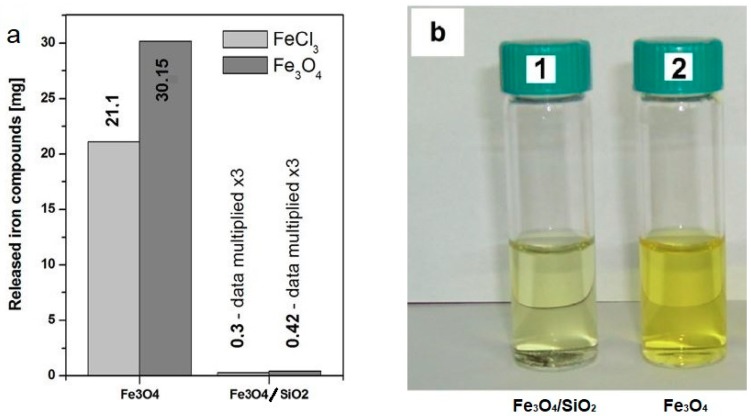
(**a**) Optical images of the concentrated hydrochloric acid after 24 h dissolution of the Fe_3_O_4_/SiO_2_ and (**b**) after extraction of undissolved nanomaterials. The samples marked 1 and 2 correspond to the Fe_3_O_4_/SiO_2_ and Fe_3_O_4_, respectively. Ultraviolet (UV)-spectroscopy-calculated amounts of the dissolved magnetite and formed iron chloride based on Cendrowski et al. (Reprinted with permission from [39]. Copyright 2017 Elsevier).

The use of a solid shell around the nanomagnetite core prevented the reaction of the acid with the core of the structure, which showed potentially high resistance to aggressive environments (Figure 3).

**Figure 4:**
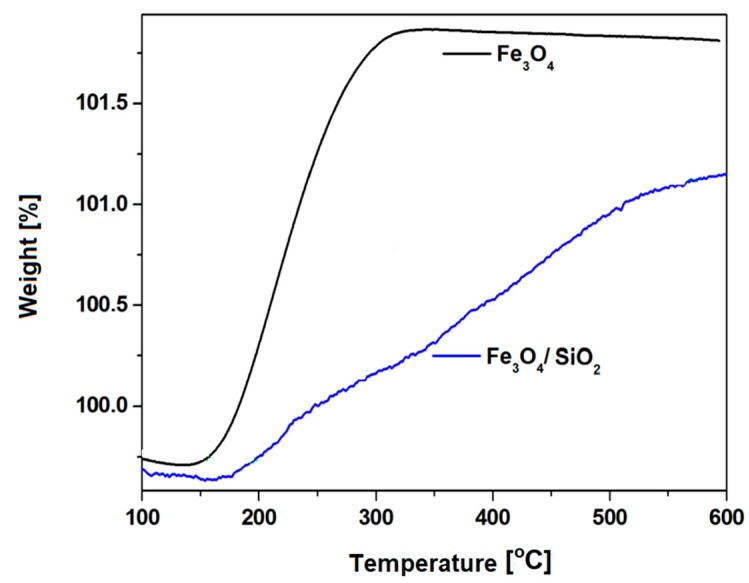
Thermogravimetric analysis of the magnetite nanoparticles (Fe_3_O_4_) and magnetite nanoparticles with a solid silica shell (Fe_3_O_4_/SiO_2_) (Reprinted with permission from [39]. Copyright 2017 Elsevier).

Thermogravimetric analysis (TGA) showed that, in the case of nanostructures covered with a solid shell, oxidation increased gradually with increasing temperature, which proves the improved resistance of the nanostructure to high temperatures (Figure 4).

The thermal stability of nano-Fe_3_O_4_ samples is dependent on many factors, including the nature of the atmosphere medium, the amount of chemical impurities, the size of the crystals, and the temperature to which the material is subjected [37,38]. de Mendonça et al. [37] studied the influence of the SiO_2_ shell on the thermal stability of nano-Fe_3_O_4_; an increase in stability was observed for the shell with an average diameter below 1 nm. In this investigation, the thermal stability of the Fe_3_O_4_/SiO_2_ samples was dependent on the nature of the atmosphere medium. In the samples after the experiment, thermogravimetric (TG) testing showed the absence of the exothermic peak at 565 °C for nano-Fe_3_O_4_/SiO_2_ particles. Moreover, the MN coating material had a less pronounced change in color (black to gray-reddish-brown), and presented an attraction to a magnet, whereas pure nano-Fe_3_O_4_ particles were fully reddish-brown with no magnetic attraction [37]. Other investigations [38,39] confirmed that the silica layer appears to be effective in protecting magnetite from being converted to other oxide species.

**Figure 5:**
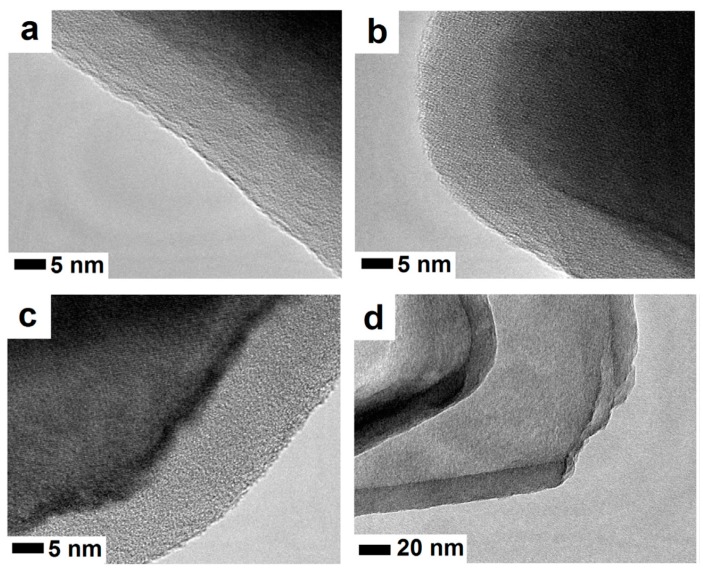
High-resolution transmission electron microscopy (HRTEM) images of the Fe_3_O_4_/SiO_2_ particle (**a**,**b**) after exposure to 550 °C and (**c**,**d**) after exposure to 550 °C and acid aggression (Reprinted with permission from [39]. Copyright 2017 Elsevier).

Fe_3_O_4_/SiO_2_ particles have shown resistance to chemically aggressive environments and when exposed to high temperatures. High-resolution transmission electron microscopy (HRTEM) analysis did not show any damage nor cracks in the shell of nanostructures heated to 550 °C (Figure 5a,b). The chemical stability of Fe_3_O_4_/SiO_2_ heated to 550 °C showed that heating the solid-silica-coated magnetite nanoparticles had no significant impact on their acid resistance. In this case, HRTEM analysis did not reveal any cracks in the solid silica coatings (Figure 5c,d). Therefore, it can be concluded that the thermal stability of the magnetite nanoparticles coated with solid silica is determined by the oxygen diffusion mechanism rather than iron oxide thermal expansion, which could cause shell cracking [39]. The use of the solid shell from the nanosilica enabled the better dispersion of the particles (Fe_3_O_4_/SiO_2_) in the cement matrix. This issue is further discussed in the next section.

## 3. Processing of MN-Engineered Cementitious Composites

**Figure 6:**
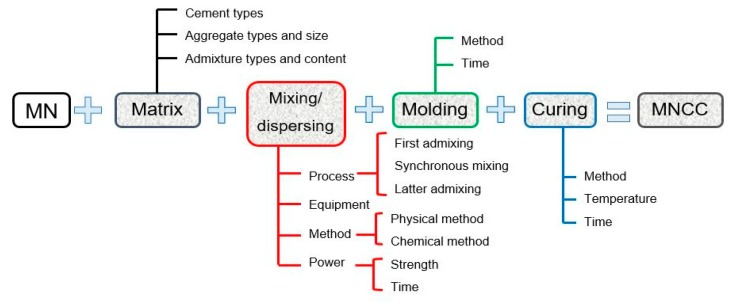
Scheme of the preparation process of magnetite nanoparticles cement composites (MNCC), based on Li et al. (Reprinted with permission from [42]. Copyright 2018 Elsevier).

Manufacturing of cement composites with magnetite nanoparticles (MN) covers preparation of the nanomodifier as well as the remaining components of the composite (cement, water, possible chemical admixtures, and additions) and their processing (e.g., mixing/dispersing, molding, and curing), as shown in Figure 6. Application of various nanoparticles, including MN, to the cement composite can cause nanomaterial agglomeration inside the cement matrix. This phenomenon is a key problem related to the practical use of NPs as admixtures in cement composites. Regarding the relatively small size of the particles and the high ratio of specific surface area to volume, the MN show a strong tendency to agglomerate [40]. The use of suitable surfactants or more sophisticated methods of introduction of the nanomaterial into the composite are necessary in such cases [41,42,43]. The occurrence of unevenly distributed nanoparticles in the cement matrix negatively affects the properties of the fresh and hardened cement composites.

Two main forms are employed for the introduction of nanosilica into a cement composite: powder or water suspension. The first method is similar to the method of introducing silica fume into concrete and mainly consists of the mechanical mixing of the cement with the NPs. Even a long mechanical mixing duration cannot ensure the proper dispersion of the nanomaterial. The results described in Kong et al. [44] show that, during mixing, the agglomerates are partially disintegrated under the action of the shear forces. The process of mixing causes a slight decrease in the number of agglomerates; however, this effect is not full and the particles are not totally broken. Thus, the development of an efficient method of dispersing the nanoparticles in a cement composite is a basic problem for many researchers, posing a significant challenge in the application of nanomaterials in the building materials industry.

For this reason, researchers more often use the second method to introduce the nanomaterial into the cement composite, involving the dispersion of the nanometric powder in the water before adding it to the dry components of the composite. In this method, ultrasonic dispersion is used in addition to mechanical mixing [45]. The nanoparticles of the magnetic compounds, besides the large specific surface area, are also magnetic, which affects the formation of agglomerates in the cement composite [33]. Sonication appears to be an effective method for dispersing the nanoparticles, including the magnetic NPs. This method, however, is energy-consuming and significantly increases the cost of application.

**Figure 7:**
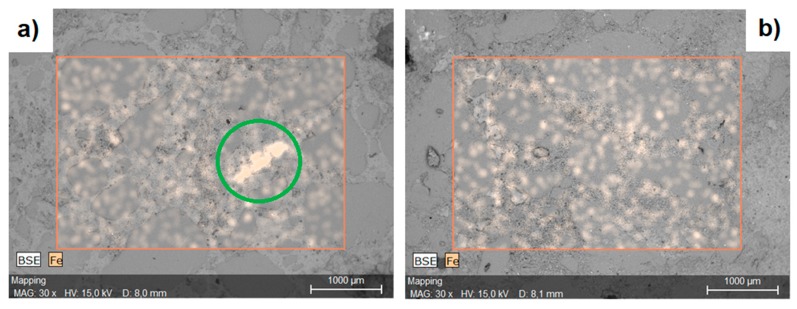
A map of Fe distribution in the cement mortar containing nano-Fe_3_O_4_: (**a**) a visible agglomerate of nano-Fe_3_O_4_ in the cement matrix; (**b**) a uniform distribution of the nanomaterial in the matrix (Reprinted with permission from [40]).

Figure 7 presents a map of Fe distribution in cement mortars modified with an admixture of nano-Fe_3_O_4_ [40]. Before the introduction of the nanomaterials into the cement mortars, the nano-Fe_3_O_4_ particles were sonicated in water for 1 min to obtain a uniform dispersion.

**Figure 8:**
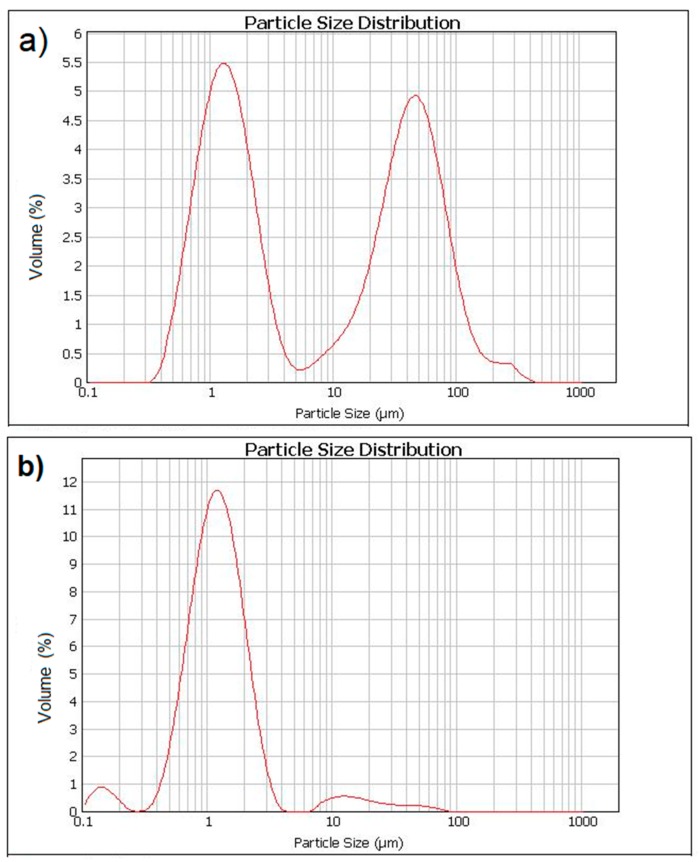
Influence of the ultrasonic dispersion (sonication) on the granulometry of Fe_3_O_4_ nanoparticles in the solution of water and superplasticizer based on polycarboxylic ether PCE: (**a**) without sonication and (**b**) after sonication for 1 min.

The impact of the ultrasonic dispersion (sonication) on the granulometry of the MN in the preparation process of the cement composite is presented in Figure 8. In the first case, the Fe_3_O_4_ NPs were mixed mechanically with a solution of water and 2% superplasticizer based on polycarboxylic ether (PCE). The agglomerates of Fe_3_O_4_ particles, which were not broken, are clearly visible in Figure 8a. Figure 8b presents the granulometric curve of the same mix after the additional process of sonication for 1 min. Incorrectly prepared dispersion of the nanomaterial and a large number of agglomerates can significantly affect the kinetics of the hydration process and, in consequence, also worsen the structure and final properties of the cement composites. The process described in Singh et al. [46], performed on the nanosilica, showed that the method of introducing the NP into the cement composite significantly influences its rheology and porosity, and thus the mechanical properties of the hardened composite. Incorrect dispersion of the NP in the cement composite can contribute to the creation of local “weak areas” with worsened mechanical features [47]. As was demonstrated by Yang et al. [48], ultrasonic dispersing can cause intensity-dependent (ultrasound frequency) breaking of the nanostructures into particles with smaller diameters.

The most often used admixtures for modern cement composites are water-reducing admixtures (plasticizers) and high-range water-reducing admixtures (superplasticizers) [40]. Superplasticizers, particularly often used for high-performance cement composites, are based on the polycarboxylic ethers [49]. The results described in Li et al. [50] confirmed that, in the case of small amounts of nanoparticles in the cement composite (below 1% of the binder mass), the use of a water-reducing admixture mechanically breaks the nanoparticles added to the previously prepared solution of water and water-reducing admixture. For nanomaterials, plasticizers and superplasticizers are surfactants that facilitate the dispersion of the nanomaterial in the composite [51].

A technique that enables the dispersion of nanoparticles in a water-reducing admixture is not yet available. Many surfactants that are successfully used to disperse nanomaterials, e.g., in polymeric matrices, have been reported to affect the cement hydration kinetics and negatively react with other admixtures. Therefore, plasticizers and superplasticizers (especially polycarboxylic ether (PCE)-based superplasticizers) compatible with cement have been widely tested and evaluated as dispersants [52,53].

**Figure 9:**
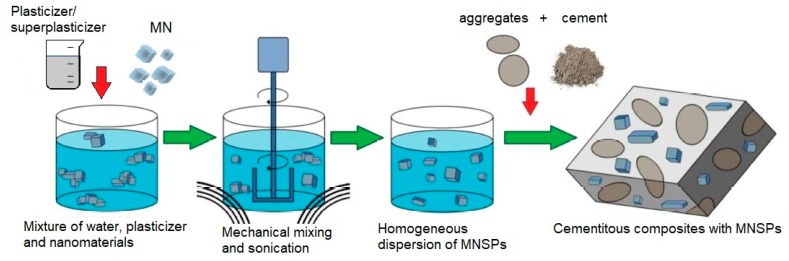
Scheme of the preparation of the cement composite containing magnetite nanoparticles (MN) based on Sikora et al. (Reprinted with permission from [51]).

Another possible solution, particularly in the case of magnetic nanoparticles, seems to be the use of an additional shell of nano-SiO_2_. Han et al. [54] demonstrated that TiO_2_ covered with a SiO_2_ nanoshell can be well-dispersed in a solution of water and water-reducing admixture using mechanical mixing. In the case of the magnetic nanostructure, the use of a nano-SiO_2_ shell also weakens the attraction forces between the particles. When large amounts of nanomaterials are used, however, ultrasonic dispersion together with mechanical stirring are necessary for ensuring the uniform distribution of the nanomaterial in the composite. Preparation of such a composite with MNPs was described in Sikora et al. [51]. To ensure the better dispersion of MN in the matrix, mechanical mixing and sonication were simultaneously used. The preparation scheme of the composite with MN used in the research described in Sikora et al. [51] and Sikora et al. [55] is presented in Figure 9.

The method of composite preparation mainly involves the selection of the method and time of compaction. An incorrect choice of the compaction method can cause additional aeration. Curing of the cement composite specimens containing MN is usually carried out in water or in a chamber with relative humidity above 95%. Additional treatments accelerating cement hydration can be applied, such as the use of hot water. The main methods for manufacturing the cement composites containing MN nanostructures are outlined in Table 2.

## 4. Properties of Cement Composites Containing Nano-Fe_3_O_4_

### 4.1. Hydration Process

**Figure 10:**
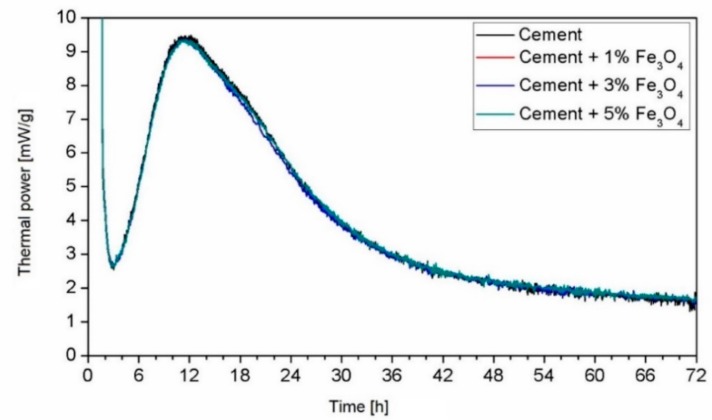
Heat flow calorimetry of the cement paste (0.5 wt %) with different dosages of nano-Fe_3_O_4_ (Reprinted with permission from [40]).

**Figure 11:**
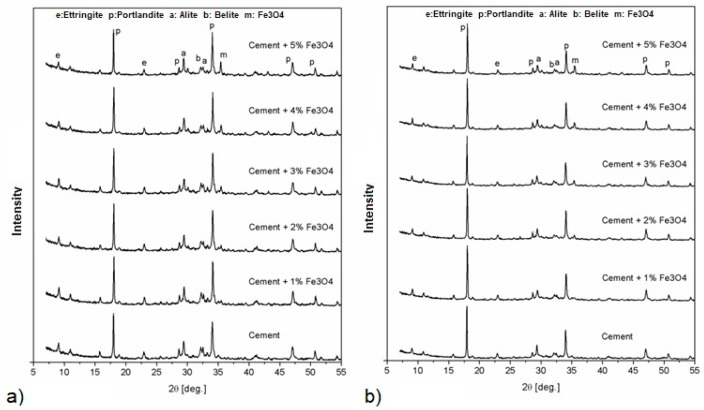
XRD spectra of the cement pastes containing nanomagnetite after (**a**) 7 and (**b**) 28 days of curing (Reprinted with permission from [41]).

The process of cement hydration is controlled not only by the mineral components of the cement particles, the size of the particles (specific surface area), the amount of the added water, and the temperature, but also by the type and content of the used nanoparticles. Studies concerning the influence of the nano-Fe_3_O_4_ admixture on the process of cement hydration are scarce. Sikora et al. [40] investigated cement pastes (Portland cement 42.5 R) modified with nano-Fe_3_O_4_ at 5% of the cement mass. The testing of the pastes during the first three days of curing did not show any changes in the hydration process (Figure 10). XRD analysis of the cement pastes containing nano-Fe_3_O_4_ in amounts from 1% to 5% of the cement mass did not reveal new hydration products in the paste after 7 days of curing (Figure 11) [40]. The main phases are calcium silicate hydrates (C–S–H), portlandite (CH), and CaCO_3_. With increased content of nano-Fe_3_O_4_ particles, the peaks related to the presence of magnetite increase. The XRD obtained after 7 days of curing displayed the same hydrate phases as the reference sample. Likewise, after 28 days of curing, no phase changes occurred. Amin et al. [60] investigated pastes containing Portland cement and high-slag cement, containing 0.1, 0.3, and 0.5% nano-Fe_3_O_4_ in relation to the cement mass. The XRD results obtained after 1, 7, and 28 days confirmed that Fe_3_O_4_ nanoparticles do not affect the rate of the cement hydration nor the characteristics of the hydration products.

### 4.2. Workability of Composites

Many investigations into cement composites modified with various nanomaterials have verified that the admixture of nanoparticles mostly negatively affects the workability of the fresh composites. Slump and slump flow are often used for the assessment of the workability of cementitious composites. A decrease in the flow with increasing nanoparticles content in the composite has been observed in many studies [61,62,63,64]. This is connected to the small size effect and large specific surface area of the nanostructures [65,66,67]. The large specific surface area and the resulting high water demand cause a reduction in the amount of free water available for the hydration process, and consequently lead to the limited workability of the cement composites [68,69].

**Figure 12:**
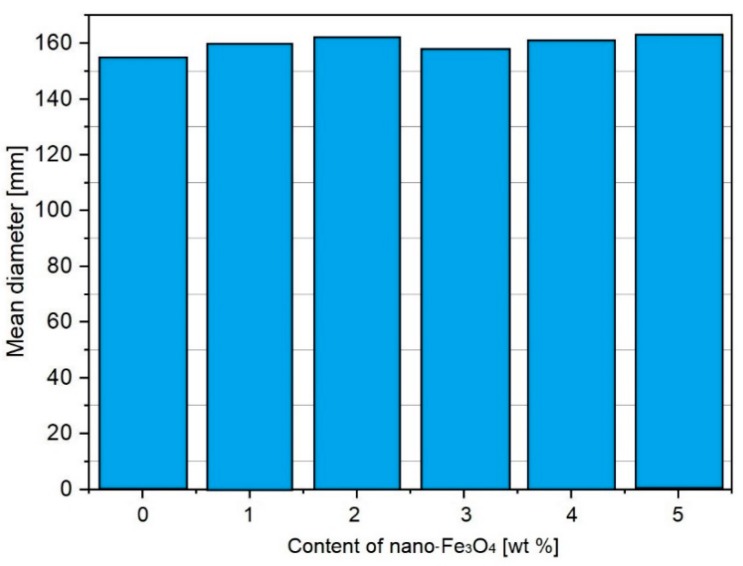
The consistency of the fresh mortars with nano-Fe_3_O_4_ (Reprinted with permission from [40]).

Most investigations into the consistency have been carried out for cement mortars modified with various amounts of MN. These tests are performed by the method according to EN 1015-3. Sikora et al. [40] tested the consistency of cement mortars containing 1–5% nano-Fe_3_O_4_ (in relation to the cement mass), without superplasticizer (water–cement ratio, *w*/*c* = 0.5 for all mortars). The test results are presented in Figure 12. The presence of nano-Fe_3_O_4_ did not significantly influence the consistency of the mortars, despite the nanometric characteristic of the modifier. This is strongly connected to the nonporous morphology of nano-Fe_3_O_4_ [70] and the more hydrophobic nature of nanomagnetite compared with other nanomaterials, such as nano-SiO_2_ or nano-TiO_2_, affecting the consistency and worsening the workability of the cement composites [46,63,64,68,71].

Bolhassani and Sayyahmanesh [56] studied the workability of mortars containing 0.05, 0.1, and 0.2% nano-Fe_3_O_4_. All mortars were designed with a low coefficient *w*/*c* = 0.28. The superplasticizer was used in amounts proportional to the content of nanomaterial (0.05%, 0.18%, and 0.55% of the binder mass, respectively). The addition of superplasticizer for obtaining a workability of mortar similar to that of the reference (without nanomodifier) appeared necessary only for mortar containing 0.2% nanomaterial.

### 4.3. Structure of Cement-Based Composites Modified with MN

**Figure 13:**
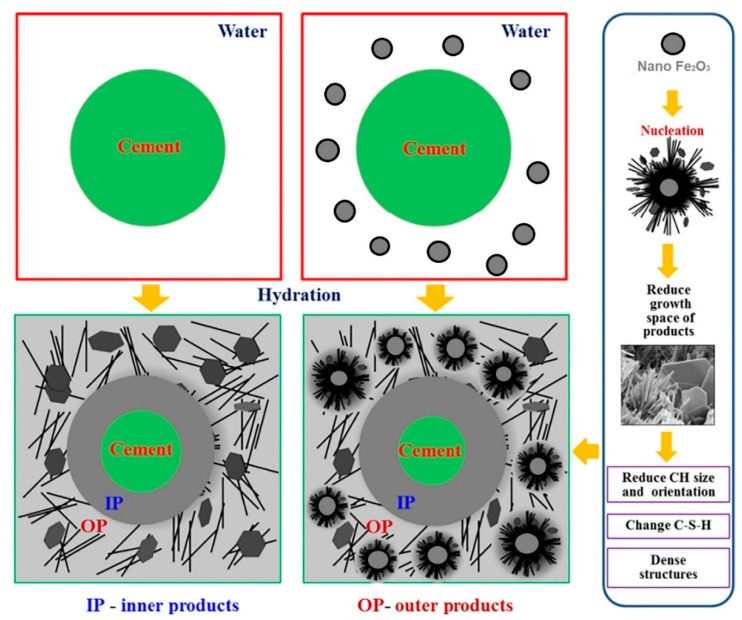
Models of influence of nano-Fe_3_O_4_ on the hydration products’ growth, based on Lee et al. (Reprinted with permission from [42]. Copyright 2018 Elsevier).

The mechanical properties of the cement composites modified with MN depend on the type, content, and form of the hydration products. Nano-Fe_3_O_4_ particles can accelerate the rate of cement hydration due to their high activity. This phenomenon has been described in Amin et al. [60], where small amounts of Fe_3_O_4_ nanometric particles were introduced into the cement paste (up to 0.3% of the cement mass). The diffusion of the hydration products starts during the hydration process and the nanometric particles are surrounded and become the crystallization nuclei around which the hydrates focus. If the amount of nanoparticles is optimum, the crystallization process will be controlled and the growth of the Ca(OH)_2_ crystals will be stopped by the nanoparticles, which in turn will improve the cement paste microstructure. However, when the amount of nanoparticles is too large, the Ca(OH)_2_ crystals cannot grow sufficiently due to the limited space in the matrix. This leads to the increase in shrinkage and creep in the cement matrix, resulting in the increased porosity of the matrix [72,73]. Moreover, the nanoparticles fill the pores due to their nanometric size and the so-called “nanofiller effect” is observed, causing further compaction of the microstructure [72]. These two phenomena lead to an improvement in the composite’s microstructure by decreasing the number of pores, strengthening the bond between the aggregate and cement paste, and increasing the density of the cement composite [72]. The scheme of the compaction process of the composite’s structure under the influence of nano-Fe_3_O_4_ is shown in Figure 13.

Nanoindentation is a method increasingly used in the investigations of the structure of the cement composites matrices on the nanoscale. Most often used is the grid indentation method or statistical nanoindentation [74,75,76,77,78]. The results of these studies led to the formulation of sophisticated models of Portland cement hydration products. The main binding phase is hydrated calcium silicate C–S–H with a heterogeneous structure. The C–S–H phase consists of 4–5 nm elementary spheres, appearing in the form of a colloid with different packing densities. Three main types (phases) of C–S–H are distinguished according to their densities: low-density (LD) C–S–H, high-density (HD) C–S–H, and the calcium hydroxide (CH)/C–S–H phase nanocomposite [79]. The strong differentiation of the hardness and Young’s modulus was observed for the particular phases when the cement composites were tested using the nanoindentation method. These results, together with statistical methods of analysis, enable the modeling of the composite nanostructure. Based on the literature data [79,80,81,82], the ranges of the Young’s modulus for the particular phases of the hardened cement pastes can be determined as follows:

(1) porous phase of low stiffness: a modulus below 10 GPa
(2) LD C–S–H phase of low stiffness: 20 ± 5 GPa
(3) HD C–S–H phase of high stiffness: 30 ± 5 GPa
(4) CH/C–S–H phase: 40 ± 5 GPa

**Figure 14:**
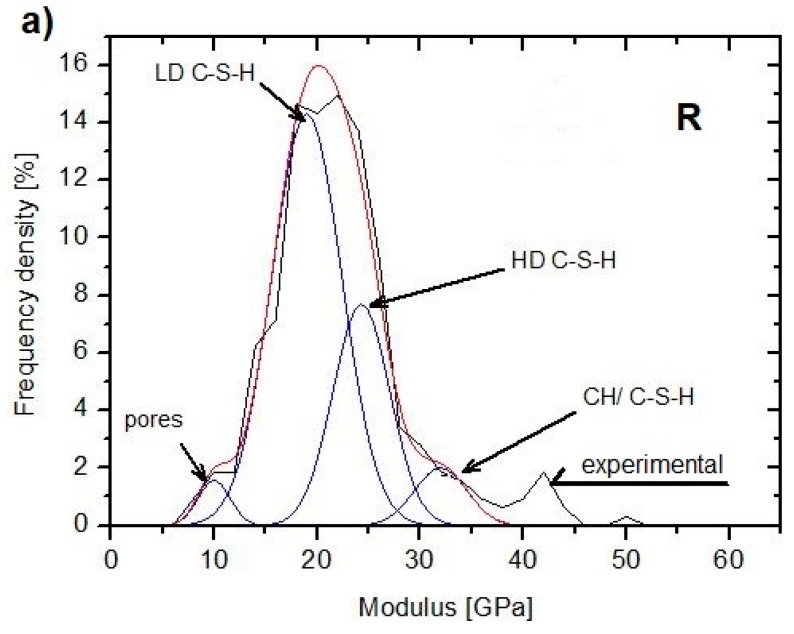

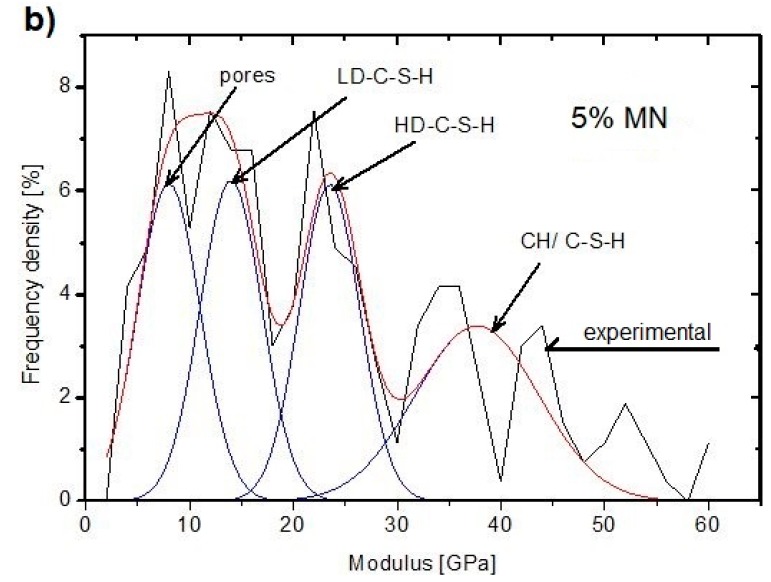
The experimental and theoretical probability distribution of the indentation modulus: (**a**) reference sample (0% nano-Fe_3_O_4_/SiO_2_) and (**b**) 5% of nano-Fe_3_O_4_/SiO_2_ (Reprinted with permission from [83]. Copyright 2017 Elsevier). LD, low density; HD, high density; CH, calcium hydroxide.

Horszczaruk et al. [83] studied cement pastes containing a 5% admixture of nano-Fe_3_O_4_ with a solid nanosilica shell, produced by the method described in Cendrowski et al. [39]. The cement pastes were produced using the Portland cement with *w*/*c* = 0.5, without a superplasticizer. Figure 14 presents the results of the nanoindentation modulus tests for the paste without the admixture (R) and with a MN admixture. Histograms of the determined Young’s modulus values were created for every specimen. Then, the obtained curves were distributed into the peaks, determining the probability of the distribution of the particular phases based on the Young’s modulus values attributed to the phases. Clearly visible is a growth in the porous phase volume in the MN sample, equal to 26.1%. Despite this increase, significant growth of the volume of the CH/C–S–H phase (25.6%) was noted compared to the reference sample (5.7%). Also, the mean values of the Young’s moduli of the CH/C–S–H phase increased by almost 20% compared to the reference sample [39]. The content of the used nanomaterial (5% of the binder mass) appeared to be significant, causing an excessive increase in the porosity of the composite. According to Cendrowski et al. [39], this phenomenon can be attributed to the large specific surface area of nanoparticles, generating higher water demand. This would confirm the investigations described in Khoshakhlagh et al. [71] and Nazari et al. [72], where the increase in porosity was explained by the lack of space for Ca(OH)_2_ crystal growth, which, in turn, leads to higher shrinkage and creep in the cement matrix. Continuing the experiment described in Horszczaruk et al. [41], Horszczaruk et al. [83] reported that a 3% admixture of nano-Fe_3_O_4_/SiO_2_ appeared to be the optimum content both in terms of the structure of the cement matrix and the final compressive strength of the composite.

The weakest link in cement composites, such as concrete or mortar, is the interfacial transition zone (ITZ), which occurs between the matrix and the aggregate or other filler. The ITZ significantly affects the macroproperties of the cement concretes, such as the strength, Young’s modulus, or crack propagation [1,2,3,84,85]. Strengthening of the transition zone is one of the reasons for the use of nanomaterials in cement composites. Such methods as SEM analysis, atomic force microscopy (AFM), or nanoindentation are employed for ITZ investigations [82,83,86,87,88,89]. The nanoindentation method or AFM technique for evaluation of the ITZ between aggregate and paste, as well as between the particular phases of the cement matrix, require the extremely careful preparation of the tested surface of the composite. As described by Horszczaruk et al. [90], the nanoindentation modulus and hardness of the cement matrix in the ITZ area of the aggregate-paste were measured. The tested objects were specially prepared specimens of the paste modified with 5% nano-Fe_3_O_4_ with a nano-SiO_2_ shell on the border of the gravel aggregate with a diameter of 10–15 mm. For this aim, the gravel grain was placed on the bottom of a 20 mm cubic moulid and flooded with the paste. After 28 days of curing, the specimen was cut to half its height and carefully polished. The details of the preparation method of the specimen surface have been described in Horszczaruk et al. [90]. The test results were compared with the reference sample made from the paste without MN admixture.

**Figure 15:**
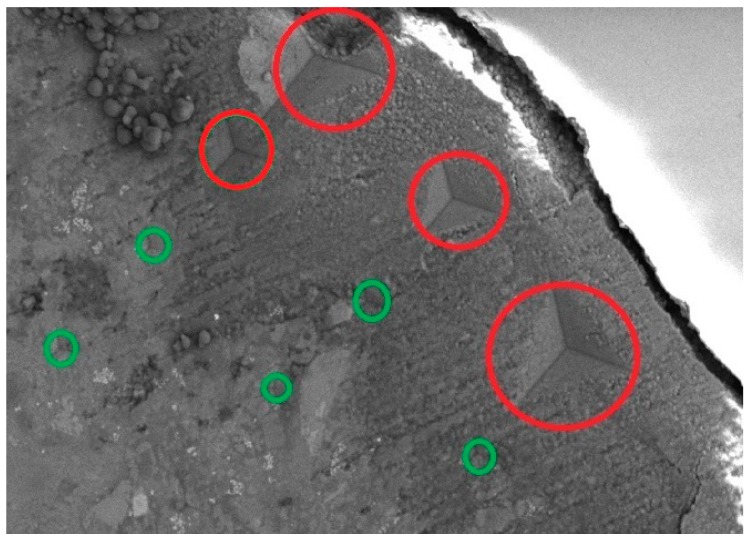
An SEM image of the imprints during the nanoindentation test (Reprinted with permission from [90]. Copyright 2018 Elsevier).

**Figure 16:**
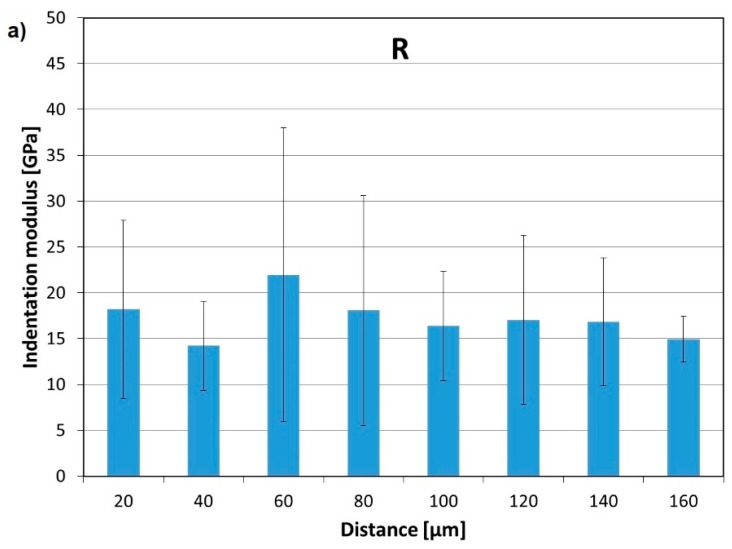

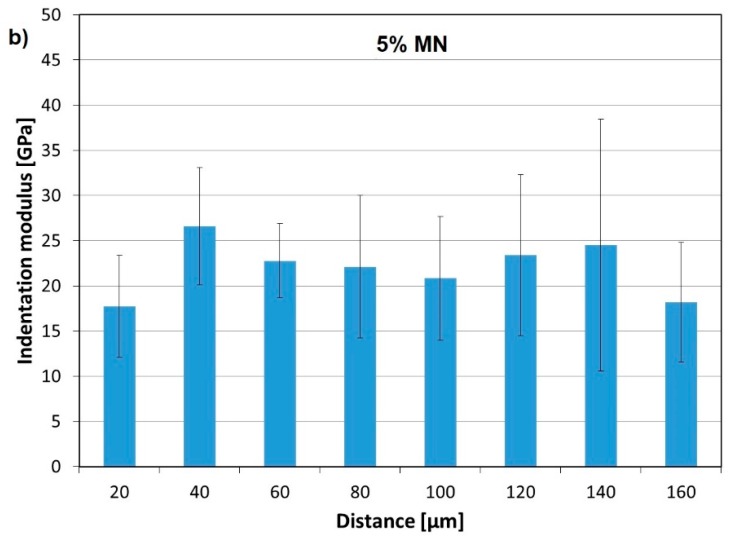
The results of measurements of the indentation modulus of the tested specimens in the transition zone: (**a**) reference sample (0% nano-Fe_3_O_4_/SiO_2_) and (**b**) 5% nano-Fe_3_O_4_/SiO_2_ content (Reprinted with permission from [83]. Copyright 2017 Elsevier).

**Figure 17:**
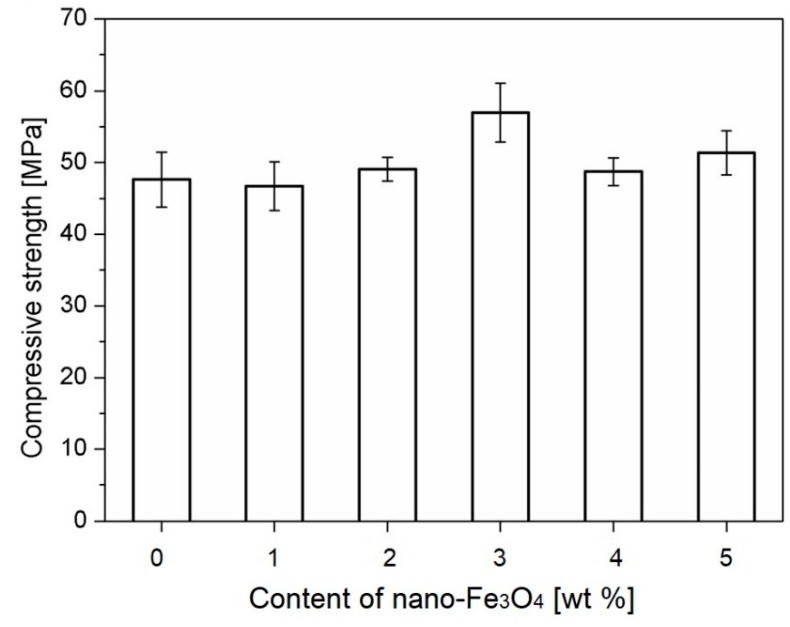
The compressive strength of cement mortars containing nano-Fe_3_O_4_ after 28 days of curing (Reprinted with permission from [40]).

The hardness and indentation modulus were measured using the nanoindentation method, using the Nanoindenter XP produced by Agilent (Santa Rosa, CA, USA) and a three-sided pyramidal Berkovich indenter. At least 15 series, each with 8 imprints in the row, were performed for every specimen. The first imprint was placed about 10–20 μm from the edge of the aggregate (Figure 17). The distance between the imprints was about 20 μm. In Figure 15, the indenter’s imprints inside the ITZ with the poorer hardness are marked in red. The imprints inside the zone with the higher hardness are marked in green. The results of testing the Young’s modulus as a function of the distance from the aggregate grain surface are presented in Figure 16.

A 13% increase in the hardness due the contact zone aggregate-paste (ITZ) and by 29% inside the cement matrix, compared to the reference composite (R), was noted for the composite containing an admixture of nanoparticles (5% MN). The mean hardness and Young’s modulus in the ITZ of the specimen containing an MN admixture were 0.59 and 17.72 MPa, respectively, and in the ITZ of the reference specimen (R), they were 0.52 and 14.27 MPa, respectively. The hardness and modulus outside the ITZ were 0.74 and 24.11 MPa, respectively, for the MN specimen, and 0.57 and 17.53, respectively, for the R specimen. A 22% increase in the value of the Young’s modulus was noted for the MN specimen in the ITZ, and 37% inside the matrix, compared with the R specimen [83]. The width of the ITZ in the specimen with 5% MN ranged from 25 to 40 μm. In the R specimen, the width of the ITZ was 40 to 70 μm. The improvement in the mechanical properties in the ITZ of the composite with NM can be explained by the use of a nano-SiO_2_ shell, which—as demonstrated in numerous studies [23,47,69,85,91,92]—accelerates the hydration process, thus enhancing the strength and characteristics of the microstructure, which leads to an improvement in the adhesion in the aggregate-paste transition zone [93].

### 4.4. Mechanical Properties

#### 4.4.1. Compressive Strength

Compressive strength is the most often investigated property of cement composites modified with MN. Only in a few papers, however, were the full test results provided, including the mean values and the standard deviations for the conducted measurements. Many authors only provide the relative strength, often presenting the data solely in graphical form, complicating comparison with the other research. The results of the compressive strength testing of the cement composites modified with MN are listed in Table 3.

Amin et al. [60] investigated the compressive strength of cement composites modified with nano-Fe_3_O_4_ at 3, 7, 14, 28, and 90 days. The pastes were prepared using Portland cement (PC) as well as cement with a high content of blast-furnace slag (75% of the slag). The pastes were modified by adding nano-Fe_3_O_4_ in the amounts of 0%, 0.05%, 0.1%, and 0.3% of the binder mass. The water-binder ratio *w*/*b* coefficient was constant at 0.30. The results showed a fast rate of hydration during the period up to 14 days with the addition of nano-Fe_3_O_4_ in PC pastes. An increase in the compressive strength of the specimens, proportional to the nano-Fe_3_O_4_ content in the paste, was observed within this period. From 28 to 90 days of curing, the specimens containing 0.05% nano-Fe_3_O_4_ showed clear growth in the compressive strength compared to the reference PC sample. Only very slight increases in the strength, lower than that of the reference sample PC, were noted for the pastes with 0.1% and 0.3% nano-Fe_3_O_4_. According to Amin et al. [60], the increase in the compressive strength in the presence of nano-Fe_3_O_4_ lower than that of the reference PC sample, especially during early hydration, could be attributed to the acceleration of the hydration reaction by nano-Fe_3_O_4_. In addition, the interaction of nano-Fe_3_O_4_ with the released free Ca(OH)_2_ led to the formation of a hydrated product with a structure similar to that of Al-ettringite, designated Fe-ettringite, which had reasonable hydraulic characteristics [60]. The Fe_3_O_4_ nanoparticles play the role of accelerators in the hydration process, which lead to an improvement in the microstructure of the modified composites.

Bolhassani and Sayyahmanesh [56] investigated cement pastes with similar composition, modified with nano-Fe_3_O_4_ and nano-Fe_3_O_4_/SiO_2_ in the amounts of 0%, 0.05%, 0.1%, and 0.2% of the binder mass, with a w/b coefficient of 0.28. They determined the compressive strength of the pastes after 3, 7, and 28 days of curing. For the pastes containing small amounts of MN (0.05%), no increase in the compressive strength was observed within the first 7 days. However, significant growth in the compressive strength within the first 7 days of specimen curing was observed for the pastes containing 0.1% and 0.2% MN. Much higher increases in the compressive strength after 7 days than after 3 days of curing were observed for all tested specimens containing MN. The greater increases in the compressive strength compared to the reference paste were noted for pastes modified with an admixture of nano-Fe_3_O_4_ than in pastes modified with nano-Fe_3_O_4_/SiO_2_, regardless of the age of the tested specimens. After 28 days of curing, all specimens with a MN admixture had higher compressive strengths than the reference paste, except for specimens with 0.2% nano-Fe_3_O_4_, which had significantly lower strength. This was caused by the poor mixing of the components in this series of specimens. Bolhassani et al. [56], similar to Amin et al. [60], attributed the increase in compressive strength to the role of magnetite nanoparticles in cores, on which the hydration products can adsorb, which, in turn, causes the compaction of the structure.

The tests of the compressive strength of the mortars modified with various amounts of nano-Fe_3_O_4_ were carried out by Sikora et al. [40]. They investigated the mortars with *w*/*c* = 0.5 containing 0%, 1%, 2%, 3%, 4%, and 5% nano-Fe_3_O_4_ in relation to the cement mass. Figure 17 shows that a small amount of nano-Fe_3_O_4_ does not significantly influence the compressive strength of the cement mortars. However, with the increase in the nanoaddition to 2 or 3 wt %, a positive effect on the compressive strength was detected. The highest compressive strength was observed for the samples containing 3 wt % nano-Fe_3_O_4_. With increasing nano-Fe_3_O_4_ to 4 or 5 wt %, however, a reduction in the strength was noticed. Therefore, the sample containing 3 wt % nano-Fe_3_O_4_ (N3) is best. These results were confirmed by the previous findings of Yazdi et al. [73] and Amin et al. [60], which showed that there is a certain amount of Fe_2_O_3_ or Fe_3_O_4_ that is beneficial for the cement composites. Exceeding this amount may result in the lowering of cement composite strength.

**Figure 18:**
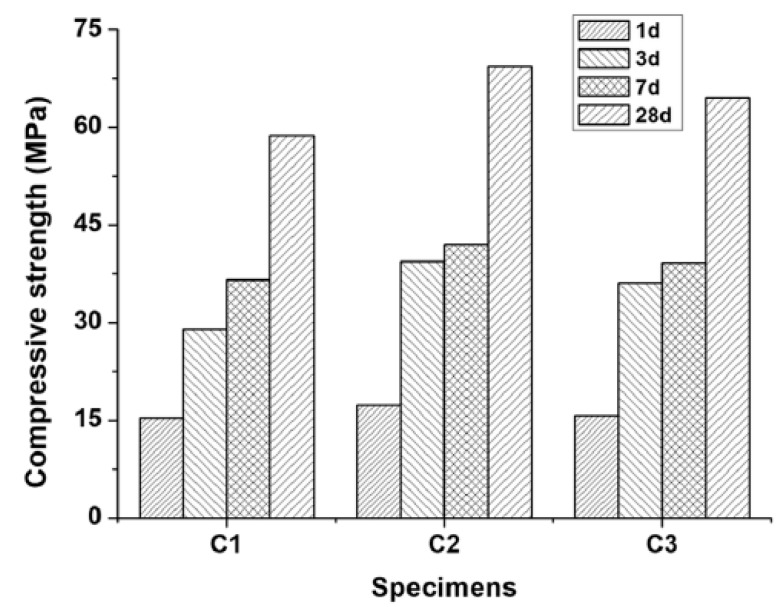
Compressive strength of the pastes: C1, reference paste; C2, paste containing nano-Fe_3_O_4_ in magnetic fluid form; and C3, paste containing nano-Fe_3_O_4_ in powder form (Reprinted with permission from [94]. Copyright 2018 Elsevier).

Investigations into the influence of the nano-Fe_3_O_4_ application method on the compressive strength of the cement paste were performed by He et al. [94]. They prepared cement paste specimens with *w*/*c* = 0.4. The C1 specimen was a reference paste, C2 contained a 5% admixture of nano-Fe_3_O_4_ in the form of a magnetic fluid, and C3 contained a 5% admixture of nano-Fe_3_O_4_ in the form of a powder. Figure 20 shows the compressive strength of the specimens. The addition of nano-Fe_3_O_4_ both as a magnetic fluid and a powder increased the compressive strength of the cement paste. The effect was more obvious for nano-Fe_3_O_4_ in the form of the magnetic fluid and during the early stages. Compared to the control specimen C1, the strength of C2 increased by 13.4%, 35.8%, and 15.0% after 1, 3, and 7 days, respectively. Similarly, the strength of C3 increased by 2.7%, 24.5%, and 7.3% after 1, 3, and 7 days, respectively. He et al. [94] explained the increase in the compressive strength in the C2 and C3 by the fact that the nanoparticles provide very large surface areas and act as nucleation sites [96,97]. The same improvement effect of nano-Fe_3_O_4_ on the strength was described by Shekari et al. [58]. As described above, the dispersion of nano-Fe_3_O_4_ in the form of a magnetic fluid in the cement paste is better than that of nano-Fe_3_O_4_ in the powder form; thus, its effect on the mechanical properties is more obvious (Figure 18).

#### 4.4.2. Flexural/Tensile Strength

Flexural strength and tensile strength are used to represent the toughness of the cement composite, which influence its brittleness. Sikora et al. [40] tested the flexural strength of the mortars containing 1–5% nano-Fe_3_O_4_. They found that the admixture of nano-Fe_3_O_4_ slightly worsened the tensile strength of the mortars (*w*/*c* = 0.5); however, this finding could be the result of the uneven distribution of the nanoparticles in the composite. In another investigation carried out by the same authors [55], the uniform distribution of the nanoparticles in the mortars was achieved using ultrasound. The mortars containing 3% and 5% nano-Fe_3_O_4_ (RF3 and RF5, respectively) and nano-Fe_3_O_4_/SiO_2_ (RFNS3 and RFNS5, respectively) were tested. The results were compared to the reference mortars containing no nanoparticles (R). The flexural strength was determined after 28 days of curing of the specimens. Similar effects in the improvement of the strength were observed for both types of the magnetite nanoparticles. Compared to the reference sample R, the flexural strength of RF3 increased by 8.5%, RF5 by 9.76%, RFNS3 by 8.5%, and RFNS5 by 12.19%.

Shekari and Razzaghi [58] investigated the tensile strength of high-strength concrete (HSC) containing a 1.5% admixture of nano-Fe_3_O_4_. They noted a 26.3% increase in the 28-day tensile strength compared to the reference concrete. Jaishankar and Mohan [59] obtained similar results from tensile strength testing for ordinary concrete with the same content of nano-Fe_3_O_4_ (1.5%). They observed an increase in the tensile strength of the specimens containing nano-Fe_3_O_4_ by about 25% compared to the reference concrete. In both investigations, the indirect method was used to determine the tensile strength of the concrete. With the limited number of studies, it is difficult to formulate specific conclusions. It can be seen, however, that the strengthening of the composite in the range of its flexural and tensile strength can be achieved, provided that the MN nanoparticles are properly distributed in the cement matrix.

### 4.5. Functional Properties

#### 4.5.1. Electromagnetic Wave Absorption

Electromagnetic waves (EMW) have found a many uses in industrial production, wireless communication, military technology, and everyday life. However, electromagnetic radiation causes environmental contamination and is potentially harmful to health; it can also be a source of noise in the transmission of information [98,99]. The negative effects of electromagnetic radiation have been a big concern, and developing electromagnetic-wave-absorbing materials is important in military and civil applications, such as stealth, microwave interference protection, and microwave darkrooms [100]. Some researchers tried to add different absorbers to cement to improve EMW absorption by the cementitious building materials [101,102,103]. The content of the absorber in the cement, necessary for efficient absorption, ranged from 10% to 30% of the mass, which significantly worsened the workability of the fresh composite and its mechanical properties. Moreover, the absorbers in powder form, used in the construction, often show a tendency of agglomerating, which further complicates their proper application in the composite.

**Figure 19:**
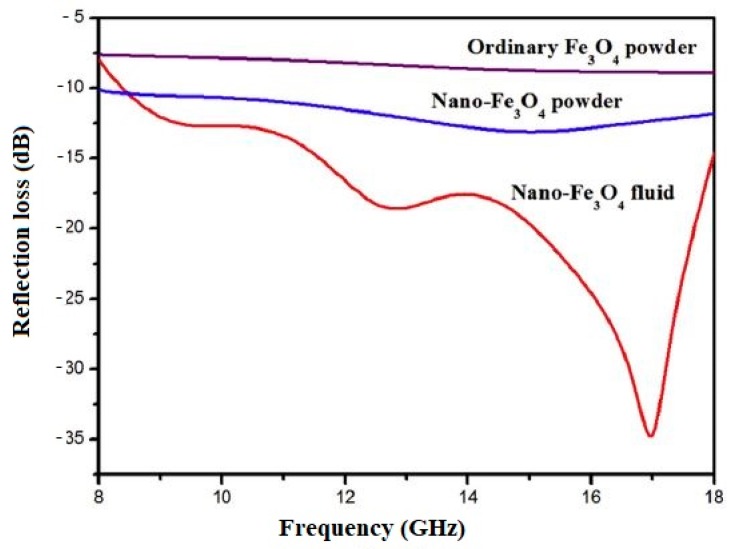
Reflection loss versus frequency for the cement composite containing various amounts of nano-Fe_3_O_4_ magnetic fluid (Reprinted with permission from [94]. Copyright 2018 Elsevier).

He et al. [94] investigated the possibility of using nano-Fe_3_O_4_ in liquid form as the admixture to enhance EMW absorption (EMWA). The nano-Fe_3_O_4_ magnetic fluid was prepared using the co-precipitation method. The obtained liquid admixture containing nano-Fe_3_O_4_ was used to prepare the cement mortars. The mortars with *w*/*c* = 0.4 and nano-Fe_3_O_4_ content equal to 3%, 5%, and 7% of the cement mass were produced. The reflection loss (RL) was determined for the characterization of the EMWA of the hardened mortars. This is an important parameter for the evaluation of EMW absorption by the materials. The more negative the RL value, the higher the MWA absorption. The RL value is affected by many factors, such as the magnetic parameters and specific surface area of the absorber, structure and thickness of the cement composite, material, and EMW frequency [42,104]. The results of tests described in He et al. [94] are presented in Figure 19. The analysis of this figure shows that the EMW reflection loss of the cement-based composite varies with the content of nano-Fe_3_O_4_ magnetic fluid. The best absorption was obtained at a mass content of 5%, at which the absorption bandwidth with RL lower than −10 dB and lower than −15 dB was about 9.5 GHz and 6.3 GHz, respectively.

**Figure 20:**
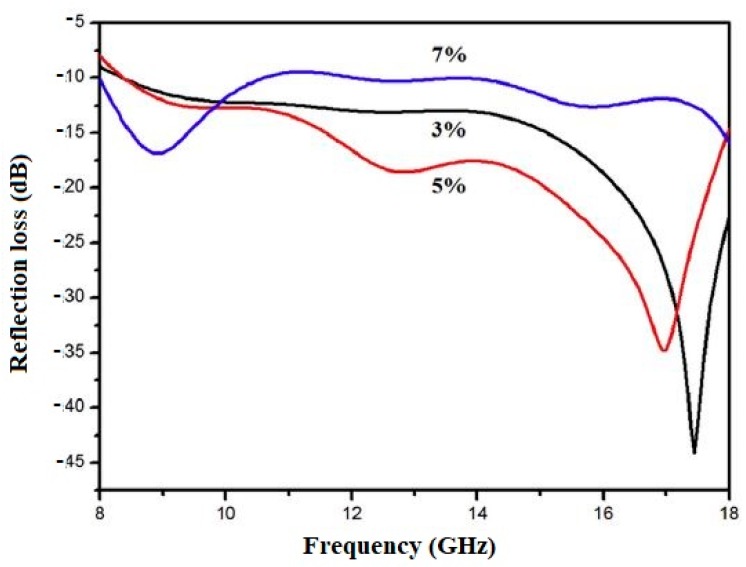
Reflection loss versus frequency for a cement composite containing 5% nano-Fe_3_O_4_ magnetic fluid, nano-Fe_3_O_4_ powder, and bulk Fe_3_O_4_ powder (Reprinted with permission from [94]).

Figure 20 shows the results of RL measurements of a cement composite with 5% nano-Fe_3_O_4_ magnetic fluid, nano-Fe_3_O_4_ powder, and bulk Fe_3_O_4_ powder. Compared to nano-Fe_3_O_4_ powder and bulk Fe_3_O_4_ powder, the nano-Fe_3_O_4_ magnetic fluid significantly lowered the RL and broadened the absorption bandwidths due to its nanoscale particle size as well as its better dispersion in the cement paste. When the size of Fe_3_O_4_ is in the nanoscale range, its electronic polarization, ion polarization, and dipole polarization are enhanced [21].

**Figure 21:**
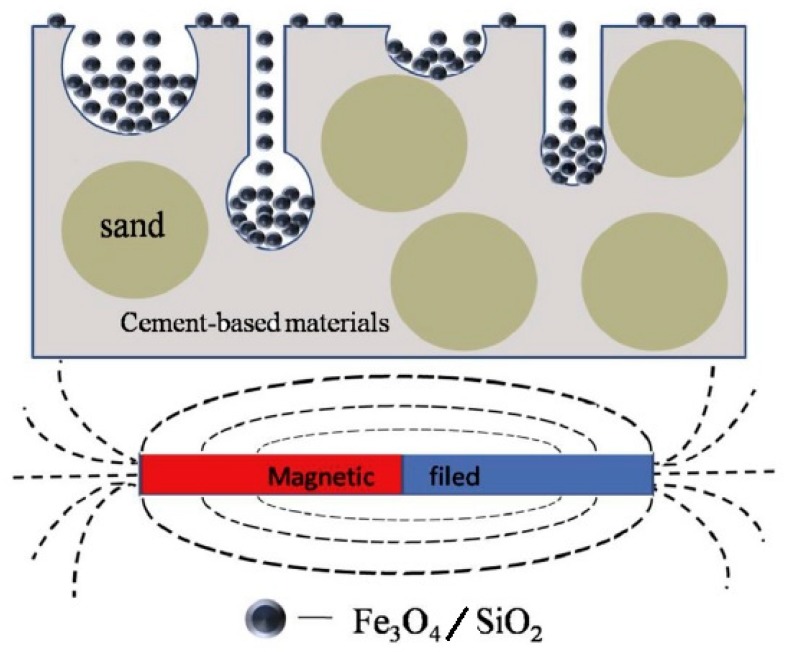
A schematic illustration of the surface treatment process of Fe_3_O_4_/SiO_2_ nanoparticles under a magnetic field (Reprinted with permission from [104]. Copyright 2017 Elsevier).

Similar results regarding the use of liquid nano-Fe_3_O_4_ as the admixture improving the EMWA of cement composites were reported by Wang et al. [104]. They used Fe_3_O_4_ nanoparticles and nanoparticles consisting of Fe_3_O_4_ with a solid SiO_2_ shell synthesized using Stöber’s method. The Fe_3_O_4_ and Fe_3_O_4_/SiO_2_ nanoparticles were dispersed in water under the same molar concentration. The mortar specimens after 28 days of curing were dried to a constant mass and then soaked using the water dispersion of the nanomaterial. The specimens’ surfaces were treated three times for 20 min. After soaking, the specimens were exposed to a magnetic field. Figure 21 provides a schematic illustration of the surface treatment process of Fe_3_O_4_/SiO_2_ particles under a magnetic field. Two hours later, the surface-treated cementitious materials were washed with water in order to remove the unbonded Fe_3_O_4_ and Fe_3_O_4_/SiO_2_ nanoparticles from the surface.

**Figure 22:**
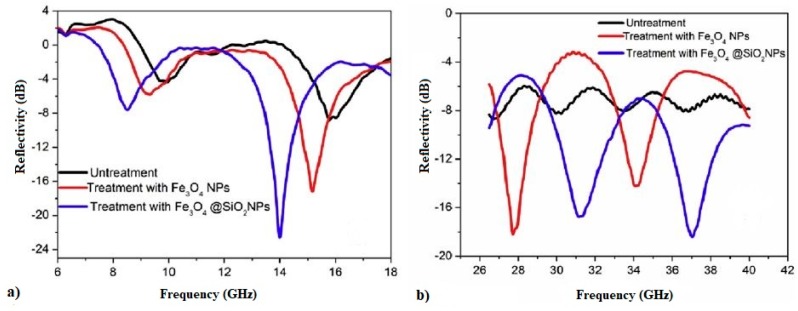
The reflectivity of cement mortar treated with Fe_3_O_4_ and Fe_3_O_4_/SiO_2_ nanoparticles (NPs) at (**a**) 6–18 GHz and (**b**) 26–40 GHz (Reprinted with permission from [104]. Copyright 2017 Elsevier).

The tests of the reflection confirmed that the use of the superficial treatment with nano-Fe_3_O_4_ and Fe_3_O_4_/SiO_2_ improved the EMWA of the tested mortars. Better results were obtained for the mortars treated with nano-Fe_3_O_4_/SiO_2_ (Figure 22).

#### 4.5.2. Gamma-Ray Shielding

A growing trend has been observed in the number of studies focused on searching for building materials that efficiently protect against gamma radiation. The basis of these studies is shielding concrete, manufactured using natural and artificial heavy aggregates [105,106,107]. Other additions to concrete that could improve its shielding properties, including waste materials, such as silica fume, barite powder, magnetite powder, fly ash, or granulated ferrous waste, have also been sought [108,109,110,111]. In this context, interest has grown in the various nanomaterials. The nanomaterials most often used for the modification of the cement composites are SiO_2_ [112], Fe_3_O_4_ [113], and PbO_2_ [114]. A resistance to high temperature is required for structural concretes exploited in nuclear power plants as well as in other objects possibly exposed to the radiation. The internal shielding layer of the concrete is often subject to heat from the reactor. For cooling water leakage from the reactor, the temperature can reach up to 360 °C. Fire only occurs in emergency or exceptional situations; therefore, the durability of structural concrete under high temperatures is very important, regardless of the object destiny. Concrete is more resistant to fire than other construction materials; however, increases in temperature above 200 °C negatively affect the mechanical properties of concrete [115,116]. High temperatures also negatively influence the shielding properties of cement concrete [117,118].

One of the main parameters enabling the evaluation of the absorption of ionizing radiation is the linear attenuation coefficient *µ*, which is the relative diminishing of intensity of a radiation beam on the unit. In the investigations into the material’s ability to absorb gamma radiation, the parameters HVL and TVL are also used. The HVL and TVL values represent the thickness of an absorber (*x*) that will reduce the gamma radiation to one-half and to one-tenth of its original intensity, respectively:

(1) $$HVL=x_{1/2}=\frac{ln2}{\mu }$$

(2) $$TVL=x_{1/10}=\frac{ln10}{\mu }$$

where *µ* is the linear attenuation coefficient (cm^−1^).

Table 4 summarizes the results of tests performed by Horszczaruk et al. [118] for cement pastes containing 5% and 10% nano-Fe_3_O_4_ in relation to the cement mass (*w*/*c* = 0.5) as well as the results of testing the cement concretes (*w*/*c* = 0.35) containing 3% nano-Fe_3_O_4_/SiO_2_ [119]. The nanomagnetite with a grain diameter of 50–100 nm produced by Sigma Aldrich (637106, Darmstadt, Germany) was used in the tests. The SiO_2_ shell synthesis method was described in detail in Cendrowski et al. [39]. The concrete specimens were made from the Portland cement CEM I 42.5 R and natural pebble aggregate up to 16 mm, with the use of the polycarboxylic superplasticizer. The pastes and concretes specimens, after 28 days of curing, were exposed to heat in a medium-temperature oven at temperatures of 300 and 450 °C. The heating method was described in Sikora et al. The linear attenuation coefficient *µ* and the values of HVL and TVL were determined for pastes specimens (RP, reference paste; P5, paste with 5% MN; and P10, paste with 10% MN) and concrete specimens (RC, reference concrete and NC, concrete with 3% MN) after heating by exposing the specimens to gamma radiation. In the investigations described in Horszczaruk et al. [118], the specimens were irradiated by a gamma ray source of ^137^Cs with an activity of 10 mCi and a photon energy of 0.662 MeV.

**Figure 23:**
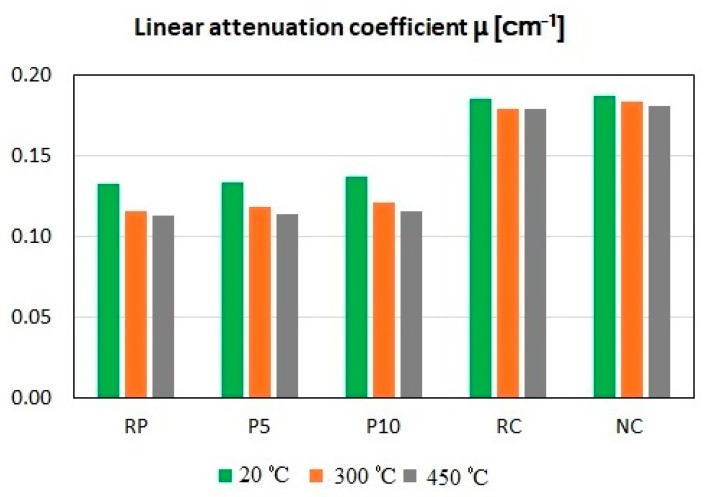
Linear attenuation coefficient *µ* for the tested cement pastes and concretes before and after exposure to 300 °C and 450 °C.

The *µ* values for the tested pastes and concretes are presented in Figure 23. The higher the *µ*, the better the shielding properties of the material. In the temperature range of 20–450 °C, both pastes and concretes with an admixture of magnetite nanoparticles showed better shielding properties than the reference composites. This was also confirmed by the results of calculation of HVL and TVL (Table 4), the values of which are lower for the concretes and pastes containing a MN admixture. As the total content of MN in the mass of the concrete is lower than 1%, the possibility of using of this type of nanoparticles in cementitious composites, particularly in repair materials, appears promising.

**Figure 24:**
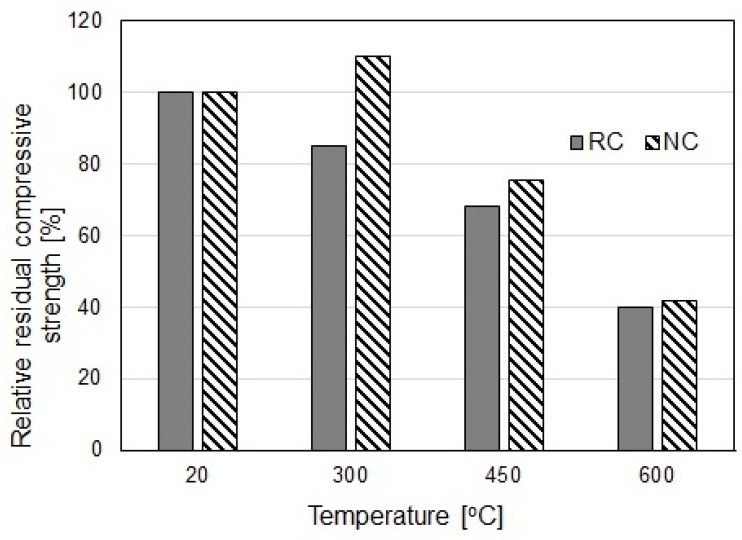
Relative changes in the compressive strength of tested concretes as a function of heating temperature (RC–reference concrete, NC–concrete with 3% of nano-Fe_3_O_4_/SiO_2_).

The value of *µ* decreases with increasing temperature; however, this decrease is small considering the relative change in the compressive strength as a function of specimen heating temperature (Figure 24). The influence of the modification of the concrete with MN particles is significant within the temperature range of 300–450 °C. At 600 °C, the destructive processes in the cement matrix begin, causing numerous cracks in the concrete and debonding of the matrix from the aggregate. This leads to a 40% decrease in the initial strength of the tested concretes.

#### 4.5.3. Thermal Resistance of Cementitious Composites

The nanoparticles of iron oxides were tested with respect to the improvement in the thermal resistance of cement composites, but few results have been published. The research described in Amer et al. [119] and Heikal et al. [120], concerning the influence of Fe_2_O_3_ nanoparticles on thermal resistance, demonstrated that even small amounts (1% of the mass) of the nanomaterial can improve the fire resistance of cement pastes due to an increase in the residual compressive strength, lower mass losses, and compaction of the composite’s microstructure. The presence of nano-Fe_2_O_3_ in cement matrices has been observed to cause a decrease in the length of the formed cracks [120]. The influence of a 5% admixture of nano-Fe_3_O_4_ on the thermal resistance of cement pastes was analysed by Mijowska et al. [120] within a temperature range of 200–800 °C. At 450 °C, the specimens containing MN were demonstrated to be about 97–100% as strong as initially, whereas the unmodified specimens were only 80% as strong. At 600 °C, the strength of the specimens containing MN and the unmodified specimens were 59–64% of the initial strength, and at 800 °C, they were 25–30% as strong. The cracking of the surfaces of the pastes modified with MN, heated at 450 and 600 °C, was reduced compared to the unmodified pastes.

However, as described in Section 2, nano-Fe_3_O_4_ is oxidized at higher temperatures, thus losing some of its properties, such as the ability to shield EMWA [121]. For this reason, various methods of improving nano-Fe_3_O_4_ stability were examined, for example, by synthesizing the nanosized protective layers on the surface of particles [37,38,39,56]. Incorporation of molecular hybrids into cement-based composites allows for the combination of the positive influences of several nanomaterials due to the synergic effect, leading to the simultaneous improvement in various properties. This effect was achieved by Bolhassani et al. [56] by incorporating the core-shell nanostructures into the cement composite. The magnetite particles acted as cores and were covered by the silica shell prepared with the Stöber’s method. This type of nanocomposite structure did not cause a significant worsening in the workability, in contrast to nanomaterials introduced separately [56]. The use of a magnetite-silica nanocomposite caused a significant increase in the relative compressive strength compared to the unmodified material [55,120].

**Figure 25:**
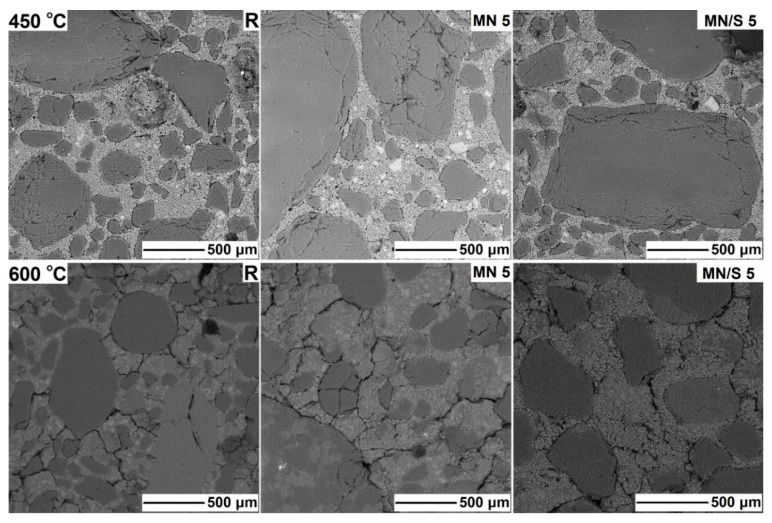
SEM micrographs of cement mortars containing 5% nano-Fe_3_O_4_ (MN 5), 5% nano-Fe_3_O_4_/SiO_2_ (MN/S 5), and reference specimen (R) exposed to 450 °C (**top**) and 600 °C (**bottom**) (Reprinted with permission from [55]. Copyright 2018 Elsevier).

The results of thermal resistance tests of the cement mortars modified with nano-Fe_3_O_4_/SiO_2_ were described in Sikora et al. [55]. The cement mortars contained 3% and 5% nano-Fe_3_O_4_/SiO_2_. The specimens were heated to 200–800 °C. The obtained results were compared with those obtained for the specimens modified with the same amounts of nano-Fe_3_O_4_ and without the nanoadmixture. The authors reported that Fe_3_O_4_/SiO_2_ nanoparticles improve the thermal resistance more by decreasing the surface crack formation in cement mortars (except at 800 °C, where the total degradation of the cement structure occurred) than the pristine Fe_3_O_4_ nanoparticles. Fe_3_O_4_ improves the thermal resistance of cement mortars at 450 °C and 600 °C (Figure 25). At lower temperatures (200 and 300 °C), the effect is negligible.

**Figure 26:**
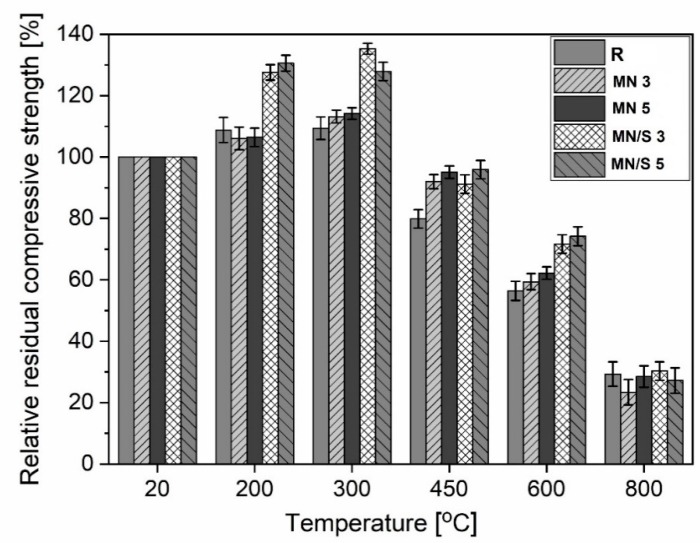
Relative residual compressive strengths of cement mortars as a function of temperature (R, reference; MN 3 and MN 5, cement mortars containing 3% and 5% nano-Fe_3_O_4_, respectively; MN/S 3 and MN/S 5, cement mortars containing 3% and 5% nano-Fe_3_O_4_/SiO_2_, respectively) (Reprinted with permission from [55]. Copyright 2018 Elsevier).

The thin silica shell (in the investigation described in Sikora et al. [55], its average thickness was 20 nm) is capable of extending the temperature range by positively influencing the effect of the iron oxide nanoparticles on the cement mortars’ residual compressive strength. The pristine Fe_3_O_4_ nanoparticles significantly improve the residual compressive strength only at 450 °C. The cement mortars containing Fe_3_O_4_/SiO_2_ exhibited improved residual compressive strength within the temperature range of 200–600 °C (Figure 26).

## 5. Concluding Remarks and Research Needs

Among all published research on the modification of cement-based composites with various nanomaterials, few studies have examined the use of nano-Fe_3_O_4_ so far. There is a common conviction that the very high cost of nanomaterials is still an insurmountable barrier for their real-world implementation in the production of cement-based building materials. Besides the prices of the nanomaterials, necessary initial investments are required that significantly affect the production costs, since the equipment and technology is expensive due to the complexity of the facilities required for the preparation of these products. Despite the basic economic limitations, certain applications of the investigations have been implemented in the building materials industry, including nano-TiO_2_ and nanosilica. The search for new materials, which could replace cement and limit CO_2_ emissions, is a worldwide trend in the building materials engineering and production field. It is important to look for new solutions despite the current lack of economic justification for the use of nanomaterials in the construction and production of building materials.

The overview of the tests results of the modification of cement composites with a MN admixture allowed for the formulation of the following conclusions:

(1) For nano-Fe_3_O_4_ used in cement composites for the modification of the structure and mechanical properties, the obtained results were similar to those recorded for the TiO_2_ and SiO_2_ nanoparticles or other nanomaterials, such as Fe_2_O_3_, Al_2_O_3_, GO, carbon nanotubes, and nanoclay. The use of MN in cement composites leads an increase in the compressive strength. However, an influence of MN on the flexural strength was not observed. MN does not show any noticeable chemical activity, and its positive impact on the mechanical properties and durability is mainly the result of the nucleation effect as well as improvement in the microstructure caused by a nanofilling effect.
(2) The obvious advantage of nano-Fe_3_O_4_ compared with other nanomaterials used for modification of the cement composites is that this addition does not worsen the workability of the composites (if its content does not exceed 10% of the binder mass). This is related to the nonporous morphology and more hydrophobic characteristics of the nanomagnetite compared to other nanomaterials, such as SiO_2_ or TiO_2_.
(3) Among the properties of the cement composites modified with MN, the particularly interesting property is the increased electromagnetic waves absorption ability and improvement in the shielding ability of the composites against gamma radiation. The tests of the attenuation of gamma rays demonstrated that the addition of nano-Fe_3_O_4_ improved the shielding ability of cement pastes and mortars in a range of energy allowing for their use in the future for shields in nuclear and medical objects exposed to ionizing radiation.
(4) The drawback of nano-Fe_3_O_4_ is the poor thermal stability of the nanoparticles. The improvement of this feature was achieved by the use of a nano-SiO_2_ shell. The use of core-shell-type nanostructues produced better mechanical properties in the cement composites within the temperature range of 200–600 °C. The noticeable limitation is the cracking of the heated composite specimens. The cement composites modified with nano-Fe_3_O_4_/SiO_2_ have demonstrated better shielding ability against gamma radiation at temperatures up to 450 °C compared to unmodified concretes. In the future, nano-Fe_3_O_4_/SiO_2_ could be used in cement-based repair materials for the injection of concrete covers in nuclear power plants.
(5) The main problem is, as with other nanomaterials, the efficient manufacturing of cement composites containing MN. The agglomeration, characteristic for nanoparticles in cement composites, increases with the addition MN due to its magnetite properties. As shown in the studies (which are still few), the effective dispersion of MN in cement composites occurs not only with sonication during the mechanical mixing of the components, but also in the coating of Fe_3_O_4_ nanoparticles with a nanosilica shell. The performance of the cement composite modified with MN is sensitive to dosage and dispersion of the nanomodifier, as well as the composition of the composite. Thus, it is important to develop an effective method for applying MN nanostructures into the composite, which should enable the production of such a composite not only in the laboratory but also in the real world.

The cement composites modified with MN are still facing, as with any new type of material, many challenges and problems, from developing production methods to understanding the mechanisms of shaping their structure and properties, to their final use.

## Figures and Tables

**Table 1:** A summary comparison of the synthesis methods [33].

Synthesis Method	Degree of Complication, Conditions	Reaction Temperature (°C)	Reaction Period	Solvent	Surface-Capping Agents	Size Distribution	Shape Control	Yield
co-precipitation	very simple, ambient conditions	20–90	minutes	water	needed, added during or after reaction	relatively narrow	not good	high/ stable
thermal decomposition	complicated, inert atmosphere	100–320	hours-days	organic compound	needed, added during reaction	relatively narrow	very good	high/ stable
microemulsion	complicated, ambient conditions	20–50	hours	organic compound	needed, added during reaction	relatively narrow	good	low
hydrothermal synthesis	simple, high pressure	220	hours ca. days	water-ethanol	needed, added during reaction	relatively narrow	very good	medium

**Table 2:** Processes available for the production of cement composites with MN particles.

Nano-Particles	MN Dispersion Method	Feeding Order	Mixing Method/Time	Molding	Curing	Reference
				Size (mm)	Condition/ Temperature	
Fe_3_O_4_/SiO_2_	Shear mixing	W + Sp + NP C + SWN	Stir/3 min Stir/2 min	Vibration/ 50 × 50 × 50 (compressive test)	Lime-saturated water 20 °C	Bolhassani and Sayyahmanesh [56]
Fe_3_O_4_	Shear mixing	C + NP W	Stir/30 min Stir/15 min	^___^/cylindrical mold: diameter 200, height 300 (compressive test)	_____	Florez et al. [57]
Fe_3_O_4_	Shear mixing	C + NP + M + S + G + W + Sp	_____	^___^/150 × 150 × 150 (compressive test) cylindrical mold: diameter 150, height 300 (indirect tensile test)	Water 20 ± 1 °C.	Shekari and Razzaghi [58]
Fe_3_O_4_	Shear mixing	C + NP S + G W + Sp	_____	^__^/150 × 150 × 150 (compressive test) ^__^/cylindrical mold: diameter 150, height 300 (indirect tensile test)	Water 20 ± 1 °C.	Jaishankar and Mohan [59]
Fe_3_O_4_	Ultrasonic method + Shear mixing	W + NP + Sp C + S SWN	Stir + ultrasonic/1 min Stir/1 min Stir/2 min	Vibration/ 40 × 40 × 160 (flexural and compressive test)	Water 20 ± 2 °C.	Sikora et al. [40]
Fe_3_O_4_Fe_3_O_4_/SiO_2_	Ultrasonic method + Shear mixing	W + NP + Sp C + S SWN	Stir + ultrasonic/30 min Stir/1 min Stir/2 min	Vibration/ 40 × 40 × 160 (flexural and compressive test)	Water 20 ± 2 °C	Sikora et al. [55]

C, cement; W, water; S, sand; G, gravel; Sp, superplasticizer; M, metacaolin; MN, magnetite nanoparticles; FA, fly ash; D, defoamer; SWN, water–nanomaterial suspension.

**Table 3:** The effect of MN on the compressive strength of cement composites.

Matrix Type/ Type of Nanoparticle	Enhancement						Content of MN (wt %)	References
	After 3 Days		After 7 Days		After 28 Days			
	Abs. (MPa)	Rel. (%)	Abs. (MPa)	Rel. (%)	Abs. (MPa)	Rel. (%)		
paste/ Fe_3_O_4_	43	0.00	55	3.77	74	4.22	0.05	Bolhassani and Sayyahmanesh [56]
	45	4.65	57	7.54	85	15.71	0.10	
	48	11.62	66	24.52	67	−5.63	0.20	
paste/ Fe_3_O_4_/SiO_2_	43	0.00	53	0.00	73	2.81	0.05	
	43	0.00	56	5.66	78	9.85	0.10	
	45	4.65	60	13.20	81	14.08	0.20	
paste/ Fe_3_O_4_	_____	_____	_____	_____	60	50.00	10.0	Flores et al. [57]
paste/ Fe_3_O_4_	_____ _____	35.80 24.50	_____ _____	15.00 7.30	____ ____	____ ____	5.0 (fluid) 5.0 (powder)	He et al. [94]
mortar/ Fe_3_O_4_	_____	_____	_____	_____	62.8	20.07	3.0	Sikora et al. [55]
	_____	_____	_____	_____	54.3	4.59	5.0	
mortar/ Fe_3_O_4_/SiO_2_	_____	_____	_____	_____	51.5	−1.53	3.0	
	_____	_____	_____	_____	55.5	6.12	5.0	
concrete/ Fe_3_O_4_	_____	_____	_____	_____	119	28.93	1.5	Shikari and Razzaghi [58]
concrete/ Fe_3_O_4_	_____	_____	_____	_____	64	4.92	1.5	Jaishankar and Mohan [59]
concrete/ Fe_3_O_4_	_____	_____	28.0	19.15	36.4	6.43	1.0	Bragança et al. [95]

Abs., absolute compressive strength; Rel., relative rate of increase in compressive strength.

**Table 4:** The results of testing the shielding properties of cement pastes and concretes.

Symbol of Specimen	MN Content (wt %)	*µ* (cm^−1^)			HVL (cm)			TVL (cm)		
		Temperature (°C)								
		20	300	450	20	300	450	20	300	450
RP	0	0.133	0.116	0.113	5.21	5.98	6.13	17.31	19.85	20.38
P5	5	0.134	0.118	0.114	5.17	5.87	6.08	17.18	19.51	20.20
P10	10	0.137	0.121	0.116	5.06	5.73	5.97	16.80	19.03	19.85
RC	0	0.186	0.179	0.179	3.73	3.85	3.87	12.38	12.79	12.86
NC	3	0.187	0.184	0.181	3.71	3.78	3.82	12.31	12.54	12.69

RP, reference paste; P5, paste with 5% MN; P10, paste with 10% MN; RC, reference concrete; NC, concrete with 3% MN.
